# Effect of posterior cruciate ligament on knee pressure and gap measured by an electronic sensor during total knee arthroplasty

**DOI:** 10.1186/s13018-023-03643-6

**Published:** 2023-03-07

**Authors:** Ran Zhao, Yanqing Liu, Hua Tian

**Affiliations:** Department of Orthopaedics, Engineering Research Center of Bone and Joint Precision Medicine, Ministry of Education, Peking University Third Hospital, 49 North Garden Road, Haidian District, Beijing, 100191 China

**Keywords:** Total knee arthroplasty, Posterior cruciate ligament, Electronics, Pressure

## Abstract

**Purpose:**

The purpose of this study was to evaluate the change in posterior cruciate ligament (PCL) tension by directly measuring the pressure changes in the knee joint when the ligament was released or resected during total knee arthroplasty.

**Methods:**

We prospectively analyzed 54 patients who underwent primary total knee arthroplasty (67 knees) from October 2019 to January 2022. An electronic pressure sensor was used to measure the pressure changes in the medial and lateral chambers on PCL retention, recession or resection.

**Results:**

At 0°, 45°, 90° and 120° of flexion, the total pressure in the knee joint of PCL retention was significantly higher than with PCL recession, and even higher than PCL resection. PCL recession or resection affected knee joint extension, and the medial/lateral pressure in the knee joint decreased. Pressure in the lateral compartment showed no significant change during knee flexion, whereas pressure in the medial compartment was significantly decreased, which also led to a change in the ratios of the medial and lateral pressures in the knee joint. After PCL resection, the flexion gap (90°) increased significantly more than the extension (0°) gap, while 46 cases displayed the same change in the flexion and extension gaps after PCL resection of the 67 cases.

**Conclusion:**

The PCL retained partial function after tibial recession. PCL resection affected both the flexion and extension gaps; although the average flexion gap increased more than the extension gap, the change in most cases of these two gaps was the same.

## Introduction

Total knee arthroplasty (TKA) is an effective method of treating end-stage knee osteoarthritis. There are two main types of knee arthroplasty: cruciate retaining (CR) prostheses and posterior stabilized (PS)/cruciate sacrificing (CS) prostheses. Currently, both types of prostheses have a 10-year survival rate of > 90% [5, 6, 9]. The posterior cruciate ligament (PCL) mainly functions to prevent the tibia rolling backwards during knee flexion. The CR prosthesis has certain advantages in improving proprioception, reproducing physiological knee biomechanics, restoring femoral retroversion and protecting the bone-cement interface from shear stress [1]. However, a PCL that is too tight leads to a reduction in the angle and increased pressure on the posterior margin of the tibial plateau, even leading to a lift-off sign, thereby accelerating polyethylene wear (both reduction in the flexion angle and increased pressure leads to lift-off sign) [20]. By contrast, excessive relaxation leads to an unstable flexion position and knee pain [8]. All of these would shorten the prosthesis survivalship and reduce patient satisfaction.

PCL preservation and optimal knee balancing are the keys to success when using the CR prosthesis. However, part of the PCL tibial insertion may be damaged during knee arthroplasty, and PCL release or tibial insertion recession may also be required when the PCL is too tight [19]. To correct possible instability and poor clinical effects, some researchers changed the CR prosthesis to a CS or PS prosthesis after releasing the PCL [10]; however, the necessity for this change is greatly debated. Even if the tibial insertion of the PCL was completely removed, the PCL would still be connected to the posterior joint capsule and retain some function. To our knowledge, no direct data support there being residual tension in the PCL after complete release of the tibial insertion.

The purposes of this study were to: (1) investigate joint pressure when the PCL is retained, released or resected, using an electronic sensor; (2) measure the pressure change in the compartment pressure distribution when the PCL is released or resected and (3) measure the changes in the flexion and extension gaps of the knee joint when the PCL is retained or resected.

## Materials and methods

The data were collected prospectively with the approval of our institutional review board. From October 2019 to January 2022, patients who underwent knee arthroplasty in our hospital were screened. Inclusion criteria were: (1) patients with osteoarthritis and (2) patients with genu varum, varus ≤ 25° or flexion contracture ≤ 25°. Exclusion criteria were: (1) patients with rheumatoid arthritis or traumatic osteoarthritis; (2) patients who had received a joint replacement with a metal gasket owing to severe varus deformity, flexion contracture or a bone defect; and (3) patients with a damaged PCL.

A total of 54 cases (67 knees) were included (16 male knees and 51 female knees). The patients’ mean age was 67.3 years (range: 56–79 years), and the mean angle of varus deformity was 12.2° (range: 5°–25°). The Smith & Nephew Genesis II PS (Smith & Nephew, London, United Kingdom) implant was used for all operations.

**Fig. 1:**
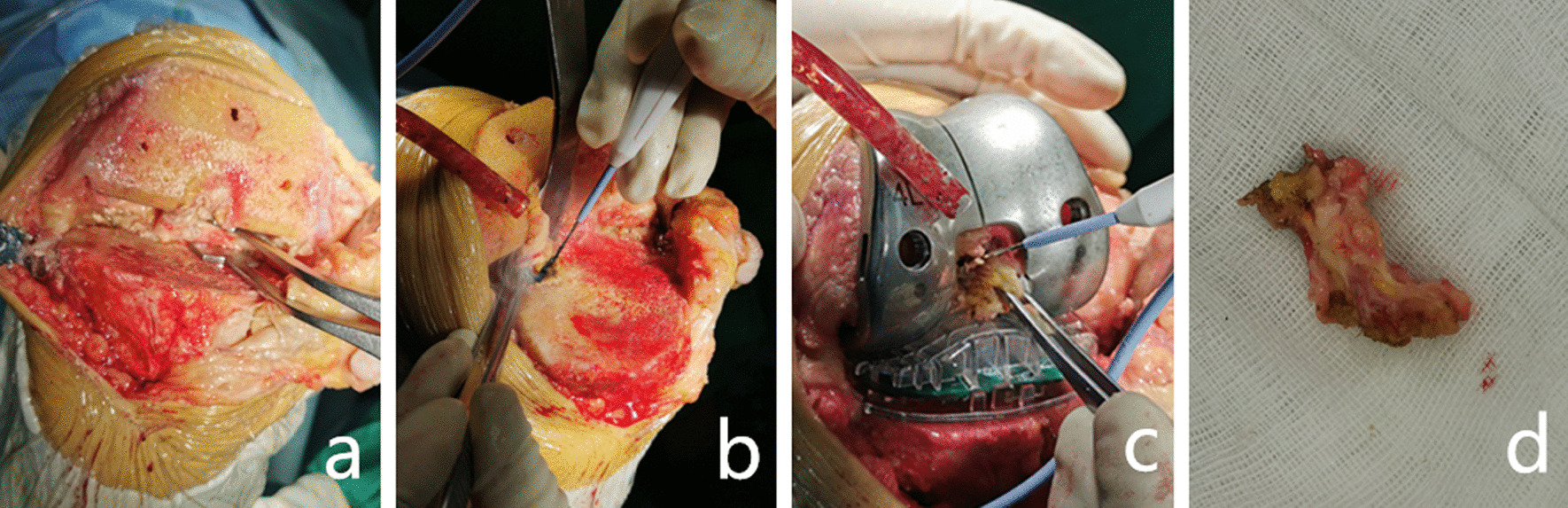
A 72-year-old female patient underwent left total knee arthroplasty for osteoarthritis of the knee **a** Following routine tibial osteotomy, attention was paid to protecting the tibial/femoral PCL insertion. **b** After obtaining the measurement data in the retention group, the femoral model and tibial sensor were removed, and the tibial insertion of the PCL was completely released to 8–10 mm below the plateau. Subsequently, the same type of femoral test model and tibial sensor were re-installed to measure the data in the tibial recession group. **c** After obtaining the measurement data in the tibial recession group, the PCL was resected, and the measurement data in the resection group were obtained. **d** Image of the resected PCL

All operations were performed by the same senior surgeon (Y. L.). A medial parapatellar approach was used for all surgeries. The deep part of the medial collateral ligament was retained and turned over the patella. The femoral resection was performed using an intramedullary guide, while the tibial resection was performed using an extramedullary guide to restore a neutral mechanical axis of the lower limb. Standard cutting blocks were used to complete the femoral and tibial preparations, a sensor was used to guide the soft tissue balancing and the tibial insertion of the PCL was retained, and the definition of soft tissue balance was an intercompartmental pressure difference between the medial and lateral compartments of 30 N or less at each flexion angle, and he pressure in the medial and lateral compartments was 0–100 N (Fig. 1a). A standard trial articular insert was used to determine the appropriate insert thickness.

**Fig. 2:**
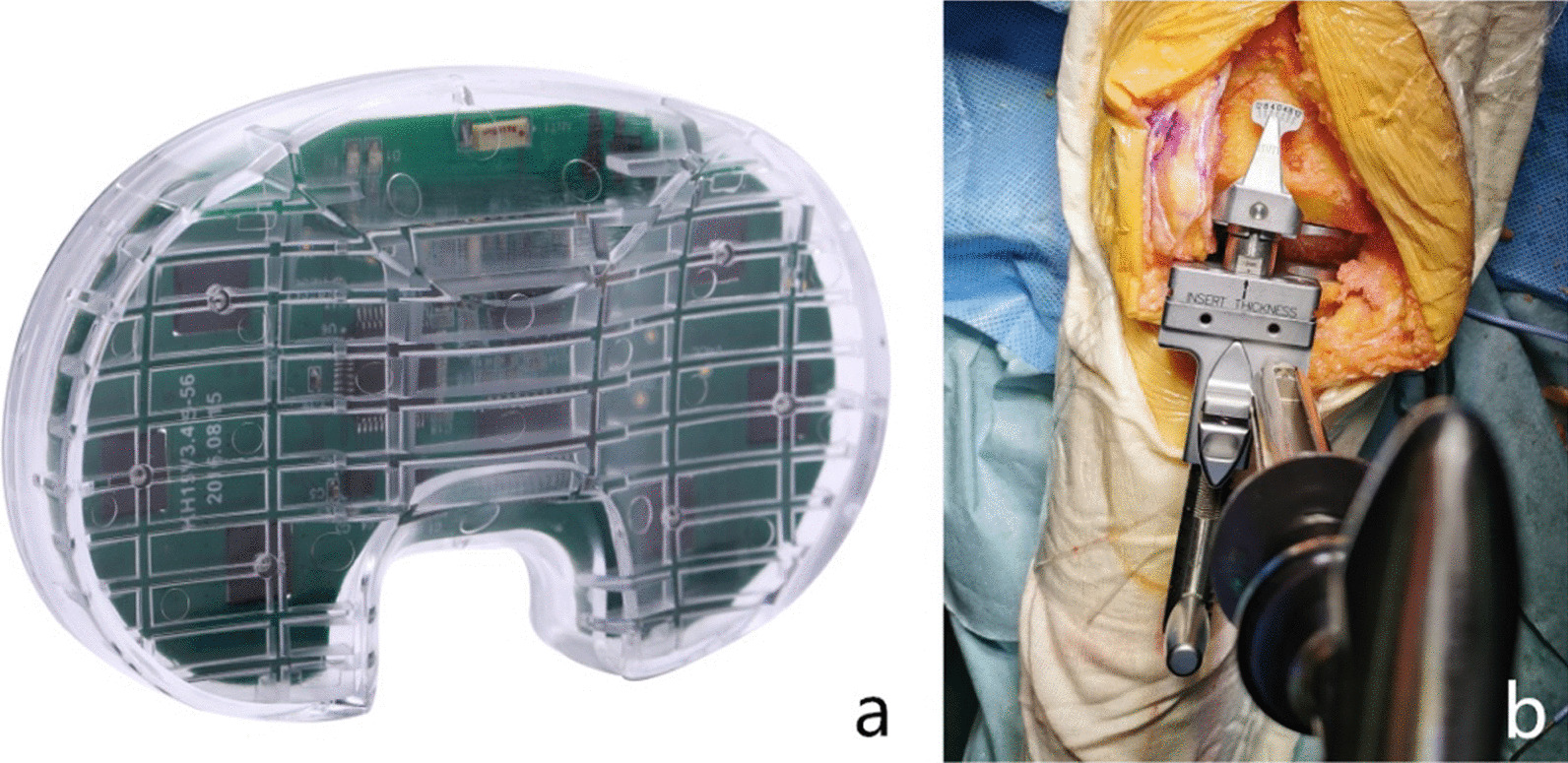
The electronic sensor and gap measurement module. **a** Image of the wireless electronic pressure sensor. **b** Image of the gap measurement module in the instrument

We used a wireless electronic pressure sensor produced by Yiemed Co. Ltd., Shandong, China (Fig. 2a), with a shape in accordance with the Genesis II CR, which can measure the pressure of the medial and lateral compartments. The electronic sensor fits gaps > 9 mm, and a 1-mm or 2-mm thickener can be used to adapt to different gaps.

The data for the PCL retention, tibial recession and resection groups were measured using the respective method described below:

1. *Acquisition of data from the retention group* After adjusting the extension-flexion gap balance, the femoral model and the matching tibial electronic sensor were placed in the knee joint space, and the medial and lateral pressures at 0°, 45°, 90° and 120° were recorded. During the measurement, the operator held the patient’s calf to ensure the foot was lifted off the operating table to counteract gravity. The sensor recorded three times at the same angle to obtain the mean value.
2. *Acquisition of data from the tibial recession group* Following the above procedure, the tibial insertion of the PCL was released to 8–10 mm below the plateau (Fig. 1b). The pressures at 0°, 45°, 90° and 120° were measured as above.
3. *Acquisition of data from the resection group* The electronic sensor and femoral model were not removed, and the femoral and tibial insertions of the PCL were completely resected (Fig. 1c, d). The pressures at 0°, 45°, 90° and 120° of flexion were measured as above.

We also measured the medial and lateral gaps at 0° and 90° before PCL recession and after PCL resection, by using the gap measurement (with 20 lbs joint distruction force) module with the minimum scale at 1 mm (Fig. 2b).

After obtaining all measurement data, the Smith & Nephew Genesis II PS-system prosthesis was used in the operation.

Statistical analysis was performed using SPSS software (IBM SPSS Statistics for Windows, Version 22.0; IBM Corp., Armonk, NY, USA). The Kolmogorov–Smirnov test was used to verify the normal distribution of the data, and the results showed that the data were non-normally distributed.

A nonparametric paired test of the total pressure, medial compartment pressure and lateral compartment pressure was conducted using the Wilcoxon signed-rank test. Using this test, we calculated the compartmental load ratio (CLR), which represented the relative load transferred through the medial compartment as follows: CLR = medial pressure/(medial pressure + lateral pressure) *100%. *P* < 0.05 was considered to be statistically significant.

Changes of the gap between 0° and 90°after PCL resection were conducted using Wilcoxon signed-rank test. *P* < 0.05 was considered to be statistically significant.

## Results

In the 67 knees undergoing TKA, the changes in total pressure in the PCL retention, recession and resection groups are shown in Table 1. The total pressure in all three groups decreased with increased flexion angle. With the knee in extension, the pressure in the retention group was the highest, at a median of 87.4 N, which decreased rapidly at 45° of flexion, with a median of 31.2 N.

**Table 1:** Pressure changes and statistical significance in the PCL retention, recession and resection groups

	Retention	Recession	Resection	Pret-rec	Pret-res	Prec-res
0° total	87.4 (69.8, 113.4)	78.5 (63.8, 102.6)	68.4 (51.8, 86.6)	0.044	< 0.001	< 0.001
0° medial	57.4 (35.2, 74.2)	40.5 (30.4, 71.3)	37.8 (26.4, 60.3)	0.006	< 0.001	0.007
0° lateral	30.0 (14.1, 44.1)	28.8 (16.0, 41.2)	21.8 (10.5, 34.9)	0.578	< 0.001	< 0.001
45° total	31.2 (23.4, 42.3)	26.8 (18.6, 38.7)	24.3 (16.8, 33.0)	0.014	< 0.001	0.081
45° medial	12.4 (5.8, 20.3)	7.9 (4.9, 13.0)	7.0 (2.4, 11.2)	< 0.001	< 0.001	0.595
45° lateral	15.8 (7.9, 22.9)	17.0 (10.2, 24.4)	13.6 (8.9, 23.7)	0.248	0.495	0.184
90° total	27.0 (17.8, 36.5)	22.9 (14.5, 34.2)	18.6 (14.7,29.5)	0.064	< 0.001	0.010
90° medial	8.7 (3.9, 13.0)	3.8 (1.9, 7.8)	2.9 (1.0, 5.0)	0.001	< 0.001	0.007
90° lateral	16.0 (7.5, 23.4)	15.1 (9.9, 23.1)	15.6 (9.9, 22.6)	0.903	0.336	0.272
120° total	27.5 (15.1. 41.3)	18.4 (10.3, 25.1)	13.3 (6.9, 25.1)	< 0.001	< 0.001	< 0.001
120° medial	8.2 (2.6, 19.3)	3.5 (1.3, 11.2)	1.7 (1.0, 7.5)	< 0.001	< 0.001	< 0.001
120° lateral	11.2 (4.1, 23.9)	9.5 (3.8, 17.5)	7.3 (2.0, 14.6)	0.033	< 0.001	0.015

*Ret* Retention, *rec* Recession, *res* Resection

Regarding the total pressure of the knee joint compartment, there were no significant differences at 45° between the recession and resection groups or at 90° between the retention and recession groups. The total pressure of the compartment was the highest on PCL retention. With the recession or resection of the PCL, the pressure of most angles decreased gradually, indicating that pressures with tibial recession of the PCL differed from those with PCL resection, and that the PCL maintained partial articular cavity pressure.

The trend of the medial compartment was similar to that for the total pressure. There was no significant difference at 45° between the recession and resection groups.

Regarding the lateral compartment pressure of the knee joint, there was no significant difference between the retention and recession groups. There was no significant difference in the lateral compartment pressure between the three groups at 45° or 90°.

The pressure in the lateral compartment exceeded the medial compartment pressure when the knee was flexed to 45°. With further release and resection of the PCL, the difference in the pressure between the medial and lateral sides of the PCL increased gradually.

**Fig. 3:**
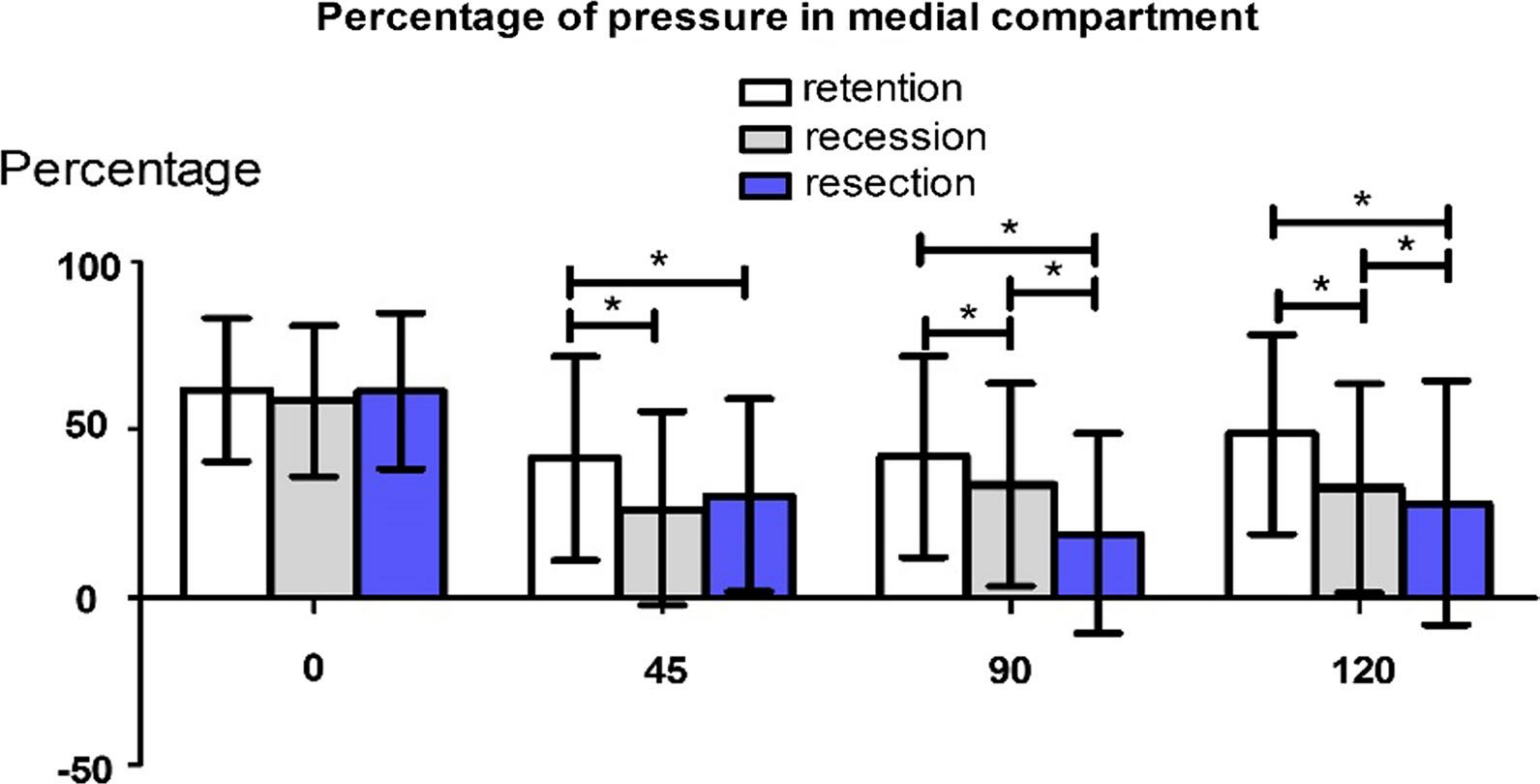
Comparison of the medial compartment pressure percentage at 0°, 45°, 90° and 120° in the three groups

The results of the medial compartment pressure percentage showed that there was no significant difference among the three groups at 0°; however, there was a significant difference among the three groups at 90° and 120°. At 45° of flexion, there was a significant difference between the retention group and both the recession and resection groups (Fig. 3).

After PCL resection, the flexion gap (90°) increased significantly more than the extension (0°) gap (*P* < 0.001). Of the 67 cases, 46 cases displayed the same change in the flexion and extension gaps after PCL resection; 18 cases displayed an increase of the flexion gap by 1 mm more than the extension gap; 3 cases displayed an increase of the flexion gap by 2 mm more than the extension gap.

## Discussion

The PCL can prevent excessive backward movement of the tibia and promote normal anatomical femoral rollback. When the knee flexion is > 90°, the PCL is stretched, and backward rolling of the femur is helpful to increase the torque in the quadriceps femoris, which improves the flexion angle of the knee joint [23]. An important measure in using a CR-type prosthesis in TKA is to balance and protect the PCL. If the PCL is too tight, it will lead to excessive femur rollback, even leading to limited knee flexion. Tightness of the PCL can also increase contact pressure and lead to polyethylene inserts wear as well as femoral lift-off, including bearing spin-out and even posterior medial dislocation of the femur [15]. Additionally, PCL release may lead to knee instability and post-operative pain [22].

### Changes of ventricular pressure after PCL retention, recession or resection

Tibial insertion recession is a method commonly used for PCL tightness. Previous literature has shown that after tibial insertion recession, the PCL remains connected to the posterior edge of the tibia through the capsule to maintain the PCL tension, and a CR-type prosthesis can be used [13]. Dion et al. [4] followed up 677 patients with a CR prosthesis for a mean of 2.5 years. They presented evidence of similar clinical outcomes when the PCL was retained or released during PCL-retaining TKA, provided attention was paid to appropriate soft tissue balancing. CR TKA undergoing partial or complete PCL release should not routinely be converted to a posterior-stabilized knee design.

Our study simulated the processes of PCL recession and resection, and the data showed that after tibial resection of the PCL, the total pressure in the knee joint decreased, which may have affected PCL function. However, the pressure in the recession group was greater than that in the resection group, indicating that the PCL still had partial tension at all angles. This finding also suggested that the PCL still functions, even if recession of the tibial insertion occurs because of osteotomy or using a Hoffman hook in TKA, which may indicate that the CR prosthesis can be used [16, 17].

Ritter et al. [16] examined 3018 CR patients, and showed that there was a minor difference in the long-term all-cause aseptic survival of both the PCL retention group (96.4% at 15 years) and the PCL recession group (96.6% at 15 years) compared with the PCL resection group (95.0% at 15 years).

### Changes of pressure distribution after PCL retention, recession or resection

In this study, the pressure in the retention group was the highest in extension (0°), and the medial pressure was greater than the lateral pressure. The pressure (particularly in the medial compartment) decreased significantly during knee flexion, particularly from 0° to 45° and then decreased gradually after 90° flexion. The lateral pressure decreased gradually and remained stable from 45° to 120°. The lateral pressure was slightly higher than the medial pressure during flexion, which was consistent with previous reports [21].

In the present study, the medial pressure and the medial compartment pressure percentage at 90° and 120° decreased significantly with PCL recession or resection, whereas the change in the lateral pressure was not significant and resulted in a higher compartment pressure percentage. A study by Iwaki et al. [7] showed that in patients who underwent CR prosthesis TKA, the medial compartment pressure percentage increased slightly with an increase in the joint flexion angle. This increase was consistent with normal knee joint anatomy reconstructed with a CR prosthesis, in which the lateral compartment rolls backwards with the medial compartment as the rotation axis. Schnaser et al. [18] used a sensor to measure the pressure in the lateral compartment in 60 cases receiving the PS prosthesis TKA and showed that with an increase in the flexion angle, the medial compartment pressure percentage decreased gradually. The pressure distribution in our results was similar to the transition from a CR to a PS prosthesis.

Our data also suggest that the PCL has a greater effect on the change in medial pressure than on lateral pressure. Following PCL resection, the medial pressure decreased significantly from 0° to 120°, whereas the lateral pressure decreased significantly only at 0°, which is consistent with the PCL maintaining the medial flexion gap and balancing valgus deformity. Therefore, PCL resection may increase the medial gap [3].

### Change of the flexion and extension gaps after PCL resection

When using the CR prosthesis, if the flexion is smaller than the extension gap, there are normally three ways to balance the gap: (1) select a smaller femoral condyle prosthesis, which allows for an increase of the osteotomy of the posterior femoral condyle; (2) increase the posterior tilt of the tibial osteotomy or (3) release or even resect the PCL to increase the flexion gap.

According to our study, although the change of the flexion gap overall was significantly greater than that of the extension gap after PCL resection, 46 of 67 cases displayed the same change between the flexion and extension gaps, indicating that PCL resection may not be a valid method for adjusting the flexion gap tension. Previous literature showed that the flexion gap after in vitro cadaveric resection of the PCL increased by 5.29 mm [12]. Matthews et al. [11] measured the gap changes after implanting a CR prosthesis followed by PCL resection, and the results showed that the extension gap increased by 0.33–0.67 mm, while the flexion gap increased by 0.53–0.66 mm. Previous studies also showed that the flexion gap increased by more than the extension gap [2, 14]. The reason for the large differences was that they used only the knee of the cadaver without the surrounding muscles. These studies applied an excessive separation force, which did not fully simulate the patient undergoing TKA.

The present study had some limitations. First, the sensor is designed for the CR prosthesis (not PS). The Genesis II CR is flatter than the PS insertion at the posterior edge to avoid blocking the rolling back of the femoral condyle. Therefore, the data after PCL resection in this study cannot fully account for the pressure changes in the PS knee joint. Second, although the sensor is sensitive to pressure, the weight of the knee and the inverting force of the operator likely affected the accuracy of the data. We attempted to balance the gravity of the knee joint to avoid potential bias; however, the position of the prosthesis also has a major impact on the intra-articular pressure. The femoral condyle prosthesis is easier to place and fix; however, when the sensor is not fixed, a different contact point between the sensor and the femur can lead to different pressures. Therefore, we marked the position of the sensor on the tibial plateau to maintain consistency for each position. Additionally, we measured the stability data three times and used the mean value to obtain accurate results. Finally, the number of cases in this study was relatively small because of the prospective data collection.

## Conclusion

The PCL retained partial function after tibial recession. PCL resection affects both the flexion and extension gaps; although the average flexion gap increased more than the extension gap, the change in most cases of these two gaps was the same.

## Data Availability

The datasets during and/or analyzed during the current study are available from the corresponding author on reasonable request.

## Abbreviations

PCL: Posterior cruciate ligament
TKA: Total knee arthroplasty
PS: Posterior stabilized
CS: Cruciate sacrificing
CR: Cruciate retaining
CLR: Compartmental load ratio
